# Definition of lines of treatment in metastatic colorectal cancer: a Delphi consensus

**DOI:** 10.1007/s12094-025-03986-y

**Published:** 2025-08-11

**Authors:** Pilar García-Alfonso, Julia Alcaide-Garcia, Enrique Aranda Aguilar, Elena Elez, Ana Fernández Montes, Ignacio García Escobar, Cristina Grávalos, Ignacio Matos García, Clara Montagut Viladot, Cristina Santos Vivas, Javier Sastre, Noelia Tarazona, Paula Jimenez-Fonseca

**Affiliations:** 1 Medical Oncology Department, Hospital General Universitario Gregorio Marañón, Instituto de Investigación Sanitaria Gregorio Marañón (IiSGM), Universidad Complutense, Madrid, Spain; 2 Medical Oncology Department, Hospital Universitario Regional de Málaga, IBIMA, Málaga, Spain; 3 Department of Medical Oncology, IMIBIC, Universidad de Córdoba, CIBERONC, Hospital Universitario Reina Sofía, Córdoba, Spain; 4 Medical Oncology Department, Vall d’Hebron Hospital Campus, Vall d’Hebron Institute of Oncology (VHIO), Universidad Autónoma de Barcelona, CIBERONC, Barcelona, Spain; 5 Medical Oncology Department, Complejo Hospitalario Universitario de Ourense, Orense, Spain; 6 Medical Oncology Department, Hospital Universitario de Toledo, Toledo, Spain; 7 Medical Oncology Department, Hospital Universitario, 12 de Octubre, Madrid, Spain; 8 Medical Oncology Department, Clínica Universidad de Navarra, Madrid, Spain; 9 Medical Oncology Department, Hospital del Mar, Hospital del Mar Research Institute, Universitat Pompeu Fabra, CIBERONC, Barcelona, Spain; 10 Medical Oncology Department, Instituto Catalán de Oncología (ICO), Translational Research Laboratory, ICO-Bellvitge Biomedical Research Institute (IDIBELL)-CIBERONC, Barcelona, Spain; 11 Department of Medical Oncology, Hospital Clínico San Carlos, Instituto de Investigación Hospital Clínico San Carlos (IdISSC), Universidad Complutense, Madrid, Spain; 12 Department of Medical Oncology, Hospital Clínico de Valencia, Valencia, Spain; 13 Department of Medical Oncology, Hospital Universitario Central de Asturias, ISPA, Oviedo, Spain

**Keywords:** Metastatic colorectal cancer, Lines of therapy, Delphi, Clinical practice guidelines, Therapeutic consensus

## Abstract

**Purpose:**

Metastatic colorectal cancer (mCRC) presents significant therapeutic challenges, with variability in the definition and classification of lines of treatment (LoTs). This study aimed to achieve consensus among Spanish oncology experts on the classification of LoTs through the application of the Delphi methodology.

**Methods:**

A nationwide Delphi study was conducted in three phases. Twelve experts designed a two-round online survey that consisted of 41 statements across 11 sections. Statements were evaluated with a five-point Likert scale, with ≥ 70% agreement or disagreement as the criterion of consensus.

**Results:**

A total of 110 and 92 oncologists participated in the first and second rounds, respectively, with consensus achieved on 32 of 41 statements. Key agreements included definition of treatment lines before systemic therapy (98.18%), classification of relapses after six months of adjuvant therapy as first line (92.73%), and the inclusion of maintenance therapy as part of first-line treatment (98.18%). Variability arose on the use of biologics in perioperative settings (67.39% disagreement) and progression criteria, and 75% of experts agreed that a switch in biologics constitutes a new line. Thus, it is needed to standardize definitions in clinical practice.

**Conclusions:**

This study highlights significant variability in the definition of LoTs for mCRC, which reflects the evolution of therapeutic landscape. The divergence between clinical trial criteria and real-world practices underscores the need for standardized definitions to enhance consistency in clinical decision-making. Refinement of guidelines on biologic agents, rechallenge strategies, and therapy classification is critical to advance mCRC management and improve patient outcomes. This consensus serves as a foundation for future research and guideline development.

**Supplementary Information:**

The online version contains supplementary material available at 10.1007/s12094-025-03986-y.

## Introduction

Colorectal cancer (CRC) is the third most common cancer and the second leading cause of cancer-related deaths globally, with approximately 1.9 million new cases each year. It begins as a benign colon or rectum adenoma that can progress to metastasis. Risk factors include age, genetics, lifestyle, and environmental factors, which make early detection and screening crucial for effective management [1–3]. Although some metastatic colorectal cancers (mCRC) are resectable, most of the cases remain incurable. Unfortunately, despite the efficacy of initial treatment lines that involve various therapeutic approaches, the majority of patients ultimately exhibit disease progression due to cancer heterogeneity and patient variability, which requires new treatment strategies. [1, 3] Several studies have indicated that the first line of treatment (LoT) is crucial for these patients, as it is generally the most effective and long lasting [4].

Recent advances in molecular profiling have provided new insights into the genetic landscape of mCRC, which highlights the importance of the identification of specific mutations such as RAS, BRAF, and microsatellite instability (MSI) status to inform treatment choices. Moreover, innovations in immunotherapy, targeted therapies, and liquid biopsy technology have an increasing influence on the management of this challenging disease and offer new avenues for more personalized treatment strategies [5].

Nevertheless, there are controversies in the definition of LoTs, as there is no official definition. This poses a problem because oncologists must determine the number of previous LoTs to dictate the optimal treatment for each patient. Additionally, this variable is essential for the establishment of inclusion and exclusion criteria in clinical trials. It is also critical to adopt a standardized approach to the definition of LoTs, both for the comparison of efficacy outcomes across different lines in real-world data registries and for the design of clinical trials and the selection appropriate endpoints. [4, 6] Consequently, the objective of this Delphi is to gather expert oncologists’ opinions in digestive tumors to reach a consensus on the definition of the different LoTs in mCRC.

## Material and methods

### Study design

This study is a nationwide exploratory study conducted by a panel of experts which followed the Delphi methodology [7]. This study consisted of two rounds, conducted from June 2024 to July 2024. A scientific board, composed of twelve expert members of the Spanish Cooperative Group for the Treatment of Digestive Tumors (TTD), participated in the design and development of the study.

The project consisted of three phases. During the first one, the preparation phase, the scientific board agreed about the design of the Delphi study and the statements to be included in the questionnaire based on the available related evidence [4, 6]. The second phase comprised a two-round survey, which was conducted online from 5th June to 16th June and from 24th June to 12th July. A scientific committee meeting discussed the results between these rounds. After the interpretation of the first-round results, the committee rephrased some statements for clarity. The second round's analysis and consensus feedback occurred in the third phase.

### Questionnaire

The questionnaire comprised 41 statements (Supplementary Table 1), grouped into 11 different sections that cover topics such as the importance of LoTs definition, the role of molecular profiling, and considerations for first-line, second-line, and subsequent therapies. It explored the influence of prior adjuvant or oligometastatic disease on treatment decisions, perioperative systemic approaches, and the selection of chemotherapy and biologic regimens. Additionally, it discussed criteria for progression, reintroduction of therapies, and the use of maintenance strategies as well as aspects related to the 3rd and subsequent lines approach. When needed, the items had a bibliographic reference attached to provide participants with additional information. The survey, anonymous and not remunerated, was sent by the TTD Group by email to all expert oncologists who are members of this scientific society. Since the process was completely anonymous, participants were advised to respond to the second round if they had participated in the first one, as the survey was distributed through an email campaign, to gather a meaningful number of responses. Out of 387, 110 participants answered in the first round and 92 answered in the second round, which represents 23.8% of the total sample of participants.

### Determination of the degree of consensus

A five-point Likert scale was used to determine the degree of consensus for each of the statements: strongly disagree (1), disagree (2), neither agree nor disagree (3), agree (4), and strongly agree (5). A consensus of agreement was established when more than 70% of the participants either “agree” or “strongly agree” for the specific item. Conversely, a consensus of disagreement was defined when more than 70% of the interviewees either “disagree” or “strongly disagree.” If neither of the two possible consensus options were met, no consensus was established for that item. Statements for which no consensus was reached after the first round were transferred to the second one, with the results from the previous round added to each item. Some of the statements from the first round were rephrased in the second round to ensure clarification and better comprehension in the interpretation of the participants.

## Results

**Fig. 1:**
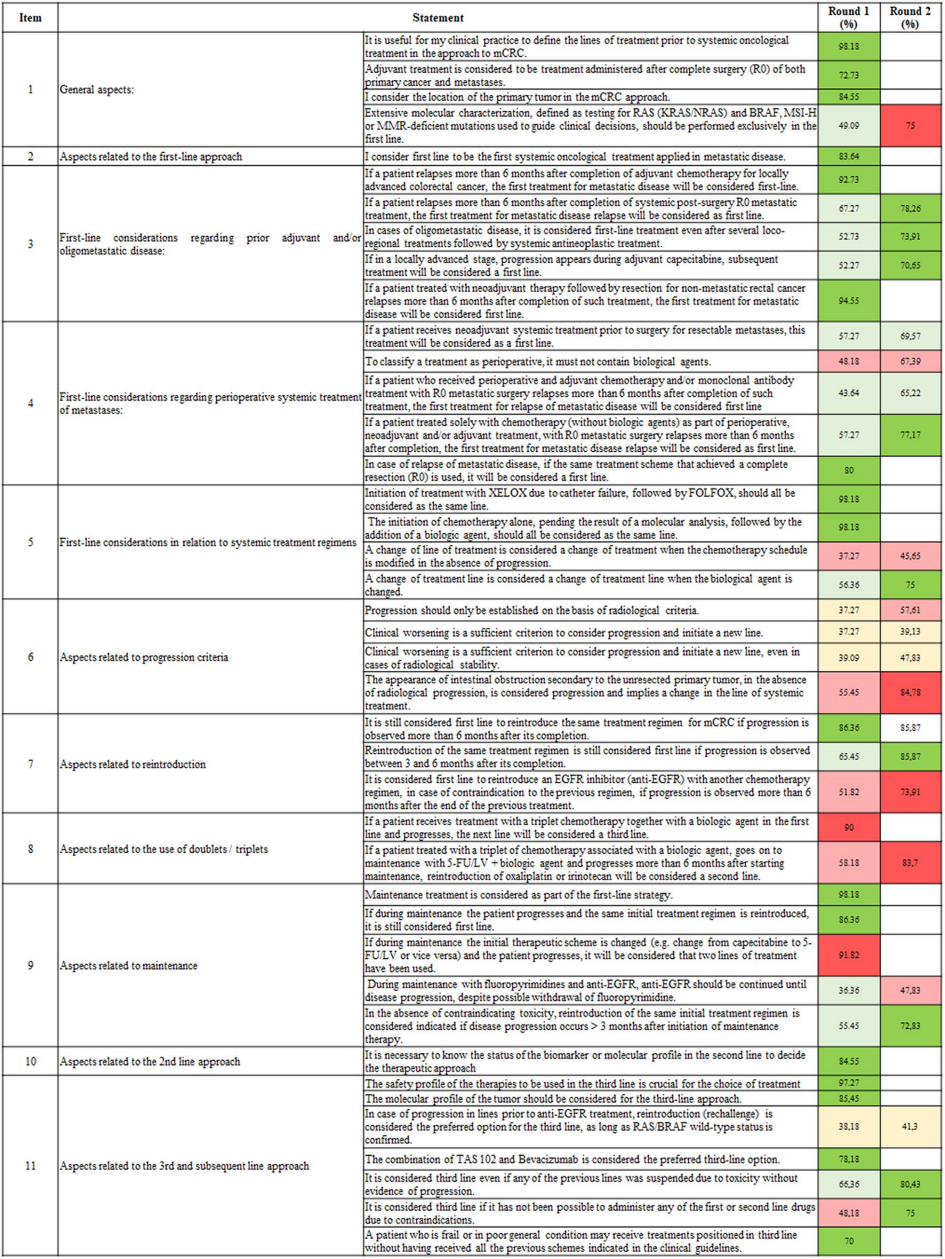
Degree of consensus for the metrics assessed in the two rounds of the Delphi method. Green represents a consensus on agreement, red represents a consensus on disagreement, and yellow represents a consensus on neither agreement nor disagreement. Light colors represent that the statement did not surpass the 70% threshold, indicating that no consensus was reached either in the overall agreement or disagreement. Conversely, strong colors represent that a consensus on the agreement or disagreement was reached.

Out of 41 statements, agreement was reached in 32, of which 19 were achieved in the first round and 13 in the second, while 9 did not achieve consensus. Results highlighted the varying levels of agreement among oncologists (Fig. 1).

In the section related to general aspects, a strong consensus was reached on the importance of LoTs definition before initiating systemic treatment in mCRC (98.2%). Similarly, most participants considered primary tumor location in treatment decision, and adjuvant treatment was widely accepted after resection of both primary cancer and metastases. However, 75% opposed the exclusive use of molecular characterization, e.g., RAS or BRAF in first line.

First-line treatment considerations had high agreement. Oncologists reached a consensus that the first systemic treatment for metastatic disease is first-line treatment. It was also considered first line in patients relapsing more than six months after adjuvant chemotherapy (92.7%) or metastatic post-surgical treatment with R0 resection (78.3%). For oligometastatic disease, 73.9% agreed systemic therapy remained first line after multiple loco-regional treatments. Treatments after progression under adjuvant capecitabine (70.65%) or relapse after neoadjuvant therapy and resection for non-metastatic rectal cancer (94.6%) were classified as first line.

Consensus was not achieved for some perioperative or neoadjuvant systemic therapy statements. The classification of neoadjuvant systemic treatment before surgery as first line reached 69.6% agreement but fell short of the consensus. Additionally, the suggestion that perioperative treatments must exclude biologic agents had 67.4% disagreement and did not reach consensus. Moreover, relapse more than six months after perioperative and adjuvant chemotherapy and/or monoclonal antibody treatments achieved 65.2% agreement, showing variability. However, consensus was reached in some scenarios. For perioperative chemotherapy (without biologic agents) followed by R0 resection, relapse after six months was considered first line (77.2%). If the same regimen that achieved R0 resection was reused post-relapse, it was also considered first line (80%). These findings underscore the variability in the definition of first-line treatments, especially in relation to the inclusion of biologics and relapse timing.

For systemic regimens in first line, consensus was high. XELOX followed by FOLFOX (98.1%) and chemotherapy initiation before molecular analysis, then the addition of a biologic agent (98.2%), were classified as the same treatment line. Conversely, no consensus was reached on whether chemotherapy changes without progression constitute a new line (45.7% disagreement), while 75% agreed switching biologics defines a new line. These results highlight agreement on clear cases but variability in nuanced situations.

Reintroduction of the same regimen in mCRC was widely supported as first line in certain contexts. A strong consensus indicated that treatments restarted more than six months after completion retained first-line classification (86.4%). The same applied to reintroductions between three and six months after initial treatment completion, which were also considered first line (85.9%). However, the use of an epidermal growth factor receptor (EGFR) inhibitor with a different chemotherapy regimen was largely rejected as first line (73.9% disagreement), considered a new line due to contraindications with prior treatment.

For doublet or triplet regimens, strong consensus opposed certain classifications. Specifically, 90% disagreed that triplet chemotherapy with a targeted agent in first line, followed by progression, should be considered third line. Similarly, 83.7% disagreed that the reintroduction of oxaliplatin or irinotecan after progression beyond six months into maintenance with 5-fluorouracil-leucovorinn (5-FU/LV) and a biologic agent constitutes second line. These results show limited support for such classifications in clinical practice.

In maintenance therapy, consensus was reached on key points. Maintenance was widely accepted as part of first-line strategy (98.1%), with reintroduction of the same regimen after progression (> 3 months) also considered first line (72.8%). Additionally, 86.36% agreed that the reintroduction of the initial regimen post-progression during maintenance remains first line. Nevertheless, 91.8% disagreed that a switch between capecitabine and 5-FU/LV in maintenance constitutes two treatment lines. No consensus was reached on continuing anti-EGFR agents until progression despite possible fluoropyrimidine withdrawal.

For second-line therapy, oncologists agreed on the necessity of biomarker or molecular profiling. Lastly, for third line and beyond, safety profiles (97.3%) and tumor molecular characteristics (85.5%) were strongly supported as key factors in therapy selection. The combination of trifluridine–tipiracil and bevacizumab was widely accepted as the preferred option (78.2%), while anti-EGFR rechallenge in RAS/BRAF wild-type tumors lacked consensus (41.3% agreed partially). Third-line classification was also endorsed for cases that involved toxicity-related treatment suspensions (80.4%) or contraindications to earlier regimens (75%). Additionally, 70% agreed that frail patients may proceed directly to third-line therapies without the completion of standard prior lines. These results emphasize flexibility and precision in the management of advanced treatment stages.

## Discussion

**Fig. 2:**
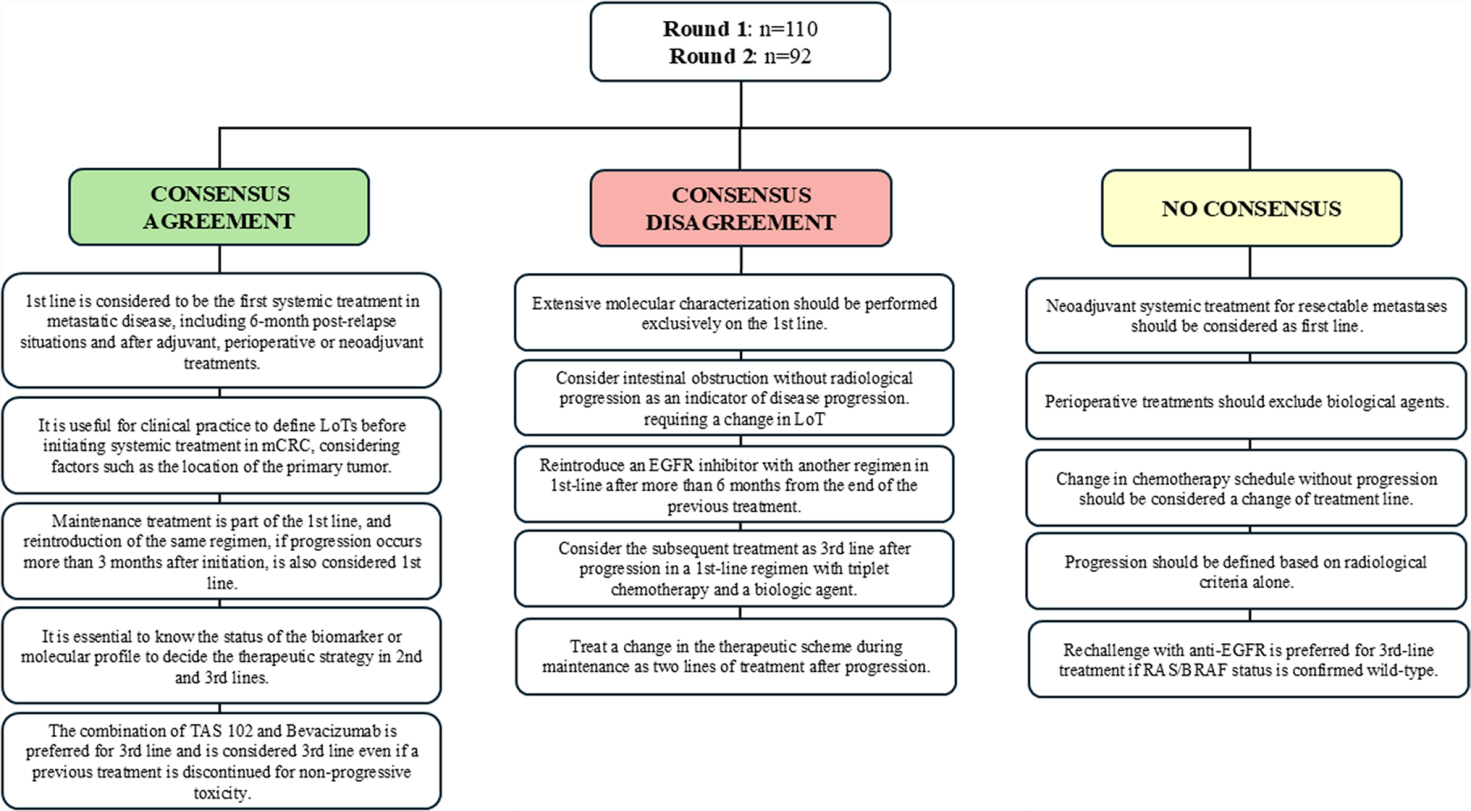
Scheme illustrating the categorization of principal ideas according to whether consensus was reached

This Delphi consensus underscores the significant variability in expert opinions and the need to refine guidelines to address the challenges identified. Future efforts should aim to unify LoT definitions that reconcile clinical trial protocols with real-world practices and clarify the role of biologic agents, progression criteria, and rechallenge strategies (Fig. 2). The resolution of these gaps will be essential for the advancement of mCRC management and the delivery of consistent, evidence-based care across diverse clinical settings.

The results highlight areas of strong agreement as well as significant variability among expert oncologists, which emphasizes the ongoing evolution in the therapeutic approaches to mCRC. These differences evidence the need for exact definitions that are adaptable to both the variability in clinical settings and the rapid advancements in mCRC treatment strategies.

Based on this analysis, a central theme that emerges is the discrepancy between real-world clinical practice and the eligibility criteria typically used in clinical trials. This divergence becomes particularly evident in the maintenance treatment setting, for example, in cases involving disease progression during adjuvant capecitabine therapy for locally advanced disease, as well as in the varying definitions of what constitutes first-line treatment. While clinical trials often classify treatments initiated within six months as part of the first-line treatment, in the clinical practice setting it is considered as a new first-line therapy any treatment after progression. [8] This disparity emphasizes the importance that has the harmonization of the definitions to ensure that clinical trials reflect real-world scenarios, which enhances their relevance and applicability. Bridging this gap would not only improve the applicability of trial results but also support the creation of universally accepted guidelines for the classification of LoTs.

To highlight the results, consensus was achieved on the reintroduction of the same treatment regimen following progression between three and six months after its initial completion, with this approach still classified as first-line therapy. Such strategies are particularly valuable in the management of patients who experience rapid progression post-surgery, as they make timely use of previously effective therapies. In relation to disagreements, there was consensus that the reintroduction of EGFR inhibitors combined with a different chemotherapy regimen after progression beyond six months could not be clearly classified. This disagreement reflects the complexities inherent in the definition of LoTs when both chemotherapy and targeted therapies are modified and underscores the need for consistent criteria. The ongoing debate in this area illustrates the need for flexibility in clinical decision-making in pursuit of standardized treatment classifications. This discussion leads to the distinction between neoadjuvant and conversion therapies, further highlighting the need of a precise classification. Neoadjuvant therapy is typically associated with curative intent, which aims to optimize surgical outcomes in resectable disease. [9] In contrast, conversion therapy is intended to downstage disease, which enables surgical resection in patients with initially unresectable metastases. [10] This ambiguity sometimes complicates the classification of these treatments within the framework of LoTs. Achievement of consensus on these definitions would not only support clinical decisions but also improve communication and interpretation of research findings. Moreover, the role of biologic agents in perioperative and relapse settings emerged as a contentious topic in the results. The conceptual confusion between neoadjuvant and conversion treatment arises again when it is determined whether a perioperative treatment should include biologics, since in resectable diseases, biologics are not used, but the interpretation of the term “perioperative” in this context leads to ambiguities that causes a lack of consensus. On the other hand, in regard to metastatic relapses after more than six months, there was consensus on the classification of relapse treatments without biologics as first line. However, the inclusion of biologics in perioperative, neoadjuvant, and/or adjuvant therapy introduced significant variability. The clinical tendency to consider biologic-inclusive regimens as different LoTs the growing importance of these agents in personalized oncology. Nevertheless, the absence of clear definitions about their use complicates their integration into treatment algorithms.

To address this ambiguity, changes in chemotherapy regimens or biologic agents also sparked debate. There was disagreement on whether a modification in a chemotherapy regimen in the absence of progression constitutes a new LoT, which reflects the variability in clinical approaches influenced by factors such as toxicity or patient preferences. This lack of consensus may stem from limited evidence in the literature. Nonetheless, there was a broad agreement indicating that switching to biological agent with a different target constitutes a new line, which aligns with guidelines that define LoT based on significant changes in therapeutic strategy [11, 12].

In addition, the definition of progression criteria was another area of variability observed. While some oncologists supported the use of clinical criteria alone to define progression, most favored combining both clinical and radiological findings. This cautious approach aims to optimize treatment decisions, minimize premature therapy transitions, and ensure that robust evidence support changes in strategy. Likewise, opinions were divided on whether complications such as intestinal obstructions caused by unresected primary tumors, in the absence of radiological progression, should prompt a change in treatment. Radiological evidence remains the preferred criterion, although it may not always capture the complexity of clinical presentations. Besides the discussion on variability in the definition of progression, the reintroduction of prior treatments, such as anti-EGFR therapies in patients with *RAS/BRAF* wild-type tumors, was another area of debate. Although clinical evidence and guidelines support the use of rechallenge strategies in specific patient populations [11, 13, 14], practical limitations, that include access to molecular testing such as liquid biopsies, pose challenges in certain settings. [15] It will be essential to overcome these logistical barriers to fully implement rechallenge approaches and maximize their potential benefit in clinical practice.

In relation to the previous point about logistical challenges, the reintroduction of oxaliplatin or irinotecan following progression after six months of maintenance therapy was widely rejected as second LoT. Thus, reintroduction is still considered part of first-line therapy, which reinforces the consistency in the classification of LoTs under specific scenarios. These findings reflect the need for clear, actionable definitions for commonly encountered clinical situations.

Furthermore, maintenance therapy received stronger agreement, with broad support for its classification as part of the first-line strategy. Moreover, the reintroduction of the same regimen after progression was considered appropriate when a sufficiently long interval had elapsed since its initial administration. This approach underscores the value in the leverage of previously effective therapies and, at the same time, taking into account the balance of time and disease dynamics [11, 14]. However, the continuation of anti-EGFR agents during maintenance was more contentious, which reflects variability in the interpretation between clinical practice and available clinical evidence, supporting the use of anti-EGFRs in maintenance, such as MACRO-2, VALENTINO, PANAMA, and ERMES studies. [16–21] Thus, further investigation is needed to define optimal maintenance strategies and ensure alignment with best practices.

To summarize, it becomes clear that while there is strong consensus on some aspects of mCRC treatment, such as the definition of LoT before systemic therapy, the classification of relapses after six months of adjuvant therapy as first line, and the inclusion of maintenance therapy as part of first-line treatment, significant debate and need for clarification persist in others. These include the use of biologics in perioperative settings and progression criteria, as well as the classification of the reintroduction of EGFR inhibitors with a different chemotherapy backbone and the continuation of anti-EGFR agents during maintenance. The ongoing dialogue among experts underscores the necessity to continually update and refine treatment guidelines based on the latest clinical research and real-world experiences, that aims to optimize patient outcomes while it adapts to the complexities and individual variations present in mCRC treatment scenarios.

In this Delphi consensus on treatment lines for mCRC, several important issues did not reach consensus, highlighting significant areas of uncertainty that require further attention. Among these is the need to clarify the classification and differentiation between neoadjuvant and conversion treatments, as well as the varied interpretations of the concept of adjuvancy in metastatic disease. The latter aspect is crucial, as it affects the classification of treatment when patients relapse after a significant period post-adjuvant therapy, which is vital for treatment planning and eligibility for clinical trials. Additionally, the use of biological agents in perioperative contexts remains controversial, which reflects a lack of agreement on their inclusion in treatment regimens. These areas of debate reflect the complexity in the establishment of uniform protocols in a field that is constantly in evolution.

This study has some limitations that should be acknowledged. Only 24% of the experts invited to participate completed the questionnaire. Nevertheless, the final sample size was considerable (n = 110) and, given the Delphi methodology and the expertise of the participating oncologists, we consider that the insights obtained are valuable for achieving a meaningful consensus.

## Conclusion

In conclusion, this consensus serves as a foundational step towards the achievement of standardized practices in the management of mCRC. The findings emphasize the importance of multidisciplinary discussions and the integration of new evidence into clinical guidelines. The gaps and ambiguities identified in this study must be addressed to advance mCRC management and ensure consistent, evidence-based care across the oncology community. Future research and efforts to reach consensus should aim to build upon these insights, which will foster greater alignment and improve outcomes for patients with mCRC.

## Supplementary Information

Below is the link to the electronic supplementary material.

Supplementary file1 (DOCX 20 KB)

## Data Availability

The authors confirm that the data supporting the findings of this study are available within the article and/or its supplementary materials.

## References

[1] Hossain MS, Karuniawati H, Jairoun AA, Urbi Z, Ooi DJ, John A, et al. Colorectal cancer: a review of carcinogenesis, global epidemiology, current challenges, risk factors. Prev Treat Strateg Cancers. 2022;14:1732. 10.3390/cancers14071732 PMC8996939 35406504

[2] Cáncer colorrectal [Internet]. [cited 2024 Dec 2]. Available from: https://www.who.int/es/news-room/fact-sheets/detail/colorectal-cancer

[3] Shin AE, Giancotti FG, Rustgi AK. Metastatic colorectal cancer: mechanisms and emerging therapeutics. Trends Pharmacol Sci. 2023;44:222–36. 36828759 10.1016/j.tips.2023.01.003 PMC10365888

[4] Modest DP, Pant S, Sartore-Bianchi A. Treatment sequencing in metastatic colorectal cancer. Eur J Cancer Oxf Engl. 1990;2019(109):70–83. 10.1016/j.ejca.2018.12.019 30690295

[5] Pathak PS, Chan G, Deming DA, Chee CE. State-of-the-art management of colorectal cancer: treatment advances and innovation. Am Soc Clin Oncol Educ Book. 2024;44: e438466. 38768405 10.1200/EDBK_438466

[6] Saini KS, Twelves C. Determining lines of therapy in patients with solid cancers: a proposed new systematic and comprehensive framework. Br J Cancer. 2021;125:155–63. 33850304 10.1038/s41416-021-01319-8 PMC8292475

[7] Shang Z. Use of Delphi in health sciences research: a narrative review. Medicine (Baltimore). 2023;102: e32829. 36800594 10.1097/MD.0000000000032829 PMC9936053

[8] Yu Q, Li Z, Liu Y, Luo Y, Fan J, Xie P, et al. Effect of different durations of adjuvant capecitabine monotherapy on the outcome of high-risk stage II and stage III colorectal cancer: a retrospective study based on a CRC database. Curr Oncol Tor Ont. 2023;30:949–58. 10.3390/curroncol30010072 PMC9857854 36661721

[9] Definición de terapia neoadyuvante - Diccionario de cáncer del NCI - NCI [Internet]. 2011 [cited 2024 Dec 2]. Available from: https://www.cancer.gov/espanol/publicaciones/diccionarios/diccionario-cancer/def/terapia-neoadyuvante

[10] Yang H, Ji K, Ji J. Current status and perspectives of conversion therapy for advanced gastric cancer. Chin J Cancer Res Chung-Kuo Yen Cheng Yen Chiu. 2022;34:109–14. 35685991 10.21147/j.issn.1000-9604.2022.02.05 PMC9086571

[11] Cervantes A, Adam R, Roselló S, Arnold D, Normanno N, Taïeb J, et al. Metastatic colorectal cancer: ESMO clinical practice guideline for diagnosis, treatment and follow-up. Ann Oncol Off J Eur Soc Med Oncol. 2023;34:10–32. 10.1016/j.annonc.2022.10.003 36307056

[12] SEOM-GEMCAD-TTD clinical guidelines for the systemic treatment of metastatic colorectal cancer (2022) - PubMed [Internet]. [cited 2024 Dec 2]. Available from: https://pubmed.ncbi.nlm.nih.gov/37133732/10.1007/s12094-023-03199-1 PMC10425293 37133732

[13] Hanovich E, Asmis T, Ong M, Stewart D. Rechallenge strategy in cancer therapy. Oncology. 2020;98(10):669–79. 32599578 10.1159/000507816

[14] Kuczynski EA, Sargent DJ, Grothey A, Kerbel RS. Drug rechallenge and treatment beyond progression–implications for drug resistance. Nat Rev Clin Oncol. 2013;10:571–87. 23999218 10.1038/nrclinonc.2013.158 PMC4540602

[15] El acceso, uno de los retos de la biopsia líquida en España con la fragmentación de centros como barrera [Internet]. [cited 2024 Dec 2]. Available from: https://gacetamedica.com/investigacion/el-acceso-uno-de-los-retos-de-la-biopsia-liquida-en-espana-con-la-fragmentacion-de-centros-como-barrera/

[16] Aranda E, García-Alfonso P, Benavides M, Sánchez Ruiz A, Guillén-Ponce C, Safont MJ, et al. First-line mFOLFOX plus cetuximab followed by mFOLFOX plus cetuximab or single-agent cetuximab as maintenance therapy in patients with metastatic colorectal cancer: phase II randomised MACRO2 TTD study. Eur J Cancer Oxf Engl. 1990;2018(101):263–72. 10.1016/j.ejca.2018.06.024 30054049

[17] Pietrantonio F, Morano F, Corallo S, Miceli R, Lonardi S, Raimondi A, et al. Maintenance therapy with panitumumab alone vs panitumumab plus fluorouracil-leucovorin in patients With RAS wild-type metastatic colorectal cancer: a phase 2 randomized clinical trial. JAMA Oncol. 2019;5:1268–75. 31268481 10.1001/jamaoncol.2019.1467 PMC6613306

[18] Morano F, Corallo S, Lonardi S, Raimondi A, Cremolini C, Rimassa L, et al. Negative hyperselection of patients with RAS and BRAF wild-type metastatic colorectal cancer who received panitumumab-based maintenance therapy. J Clin Oncol Off J Am Soc Clin Oncol. 2019;37:3099–110. 10.1200/JCO.19.01254 PMC6864846 31539295

[19] Modest DP, Karthaus M, Fruehauf S, Graeven U, Müller L, König AO, et al. Panitumumab plus fluorouracil and folinic acid versus fluorouracil and folinic acid alone as maintenance therapy in RAS wild-type metastatic colorectal cancer: the randomized PANAMA trial (AIO KRK 0212). J Clin Oncol Off J Am Soc Clin Oncol. 2022;40:72–82. 10.1200/JCO.21.01332 PMC8683209 34533973

[20] Sommerhäuser G, Kurreck A, Beck A, Fehrenbach U, Karthaus M, Fruehauf S, et al. Depth of response of induction therapy and consecutive maintenance treatment in patients with RAS wild-type metastatic colorectal cancer: an analysis of the PanaMa trial (AIO KRK 0212). Eur J Cancer Oxf Engl. 1990;2023(178):37–48. 10.1016/j.ejca.2022.09.011 36399909

[21] Pinto C, Orlandi A, Normanno N, Maiello E, Calegari MA, Antonuzzo L, et al. Fluorouracil, leucovorin, and irinotecan plus cetuximab versus cetuximab as maintenance therapy in first-line therapy for RAS and BRAF wild-type metastatic colorectal cancer: phase III ERMES study. J Clin Oncol Off J Am Soc Clin Oncol. 2024;42:1278–87. 10.1200/JCO.23.01021 PMC11095858 38181312

