# Supplementary material for: Definition of lines of treatment in metastatic colorectal cancer: a Delphi consensus
Source: Clin Transl Oncol. 2025 Aug 11;28(2):617–24. doi: 10.1007/s12094-025-03986-y (PMC12855278; doi:10.1007/s12094-025-03986-y)
Supplement: Supplementary file 1 — Supplementary file1 (DOCX 20 KB) [file 12094_2025_3986_MOESM1_ESM.docx]

| **Item** | **Statement** |
| --- | --- |
| General aspects | It is useful for my clinical practice to define the lines of treatment prior to systemic oncological treatment in the approach to mCRC. |
|  | Adjuvant treatment is considered to be treatment administered after complete surgery (R0) of both primary cancer and metastases. |
|  | I consider the location of the primary tumor in the mCRC approach. |
|  | Extensive molecular characterization, defined as testing for RAS (KRAS/NRAS) and BRAF, MSI-H or MMR-deficient mutations used to guide clinical decisions, should be performed exclusively in the first line. |
| Aspects related to the first-line approach | I consider first line to be the first systemic oncological treatment applied in metastatic disease. |
| First-line considerations regarding prior adjuvant and/or oligometastatic disease | If a patient relapses more than 6 months after completion of adjuvant chemotherapy for locally advanced colorectal cancer, the first treatment for metastatic disease will be considered first-line. |
|  | If a patient relapses more than 6 months after completion of systemic post-surgery R0 metastatic treatment, the first treatment for metastatic disease relapse will be considered as first line. |
|  | In cases of oligometastatic disease, it is considered first-line treatment even after several loco-regional treatments followed by systemic antineoplastic treatment. |
|  | If in a locally advanced stage, progression appears during adjuvant capecitabine, subsequent treatment will be considered a first line. |
|  | If a patient treated with neoadjuvant therapy followed by resection for non-metastatic rectal cancer relapses more than 6 months after completion of such treatment, the first treatment for metastatic disease will be considered first line. |
| First-line considerations regarding perioperative systemic treatment of metastases | If a patient receives neoadjuvant systemic treatment prior to surgery for resectable metastases, this treatment will be considered as a first line. |
|  | To classify a treatment as perioperative, it must not contain biological agents. |
|  | If a patient who received perioperative and adjuvant chemotherapy and/or monoclonal antibody treatment with R0 metastatic surgery relapses more than 6 months after completion of such treatment, the first treatment for relapse of metastatic disease will be considered first line |
|  | If a patient treated solely with chemotherapy (without biologic agents) as part of perioperative, neoadjuvant and/or adjuvant treatment, with R0 metastatic surgery relapses more than 6 months after completion, the first treatment for metastatic disease relapse will be considered as first line. |
|  | In case of relapse of metastatic disease, if the same treatment scheme that achieved a complete resection (R0) is used, it will be considered a first line. |
| First-line considerations in relation to systemic treatment regimens | Initiation of treatment with XELOX due to catheter failure, followed by FOLFOX, should all be considered as the same line. |
|  | The initiation of chemotherapy alone, pending the result of a molecular analysis, followed by the addition of a biologic agent, should all be considered as the same line. |
|  | A change of line of treatment is considered a change of treatment when the chemotherapy schedule is modified in the absence of progression. |
|  | A change of treatment line is considered a change of treatment line when the biological agent is changed. |
| Aspects related to progression criteria | Progression should only be established on the basis of radiological criteria. |
|  | Clinical worsening is a sufficient criterion to consider progression and initiate a new line. |
|  | Clinical worsening is a sufficient criterion to consider progression and initiate a new line, even in cases of radiological stability. |
|  | The appearance of intestinal obstruction secondary to the unresected primary tumor, in the absence of radiological progression, is considered progression and implies a change in the line of systemic treatment. |
| Aspects related to reintroduction | It is still considered first line to reintroduce the same treatment regimen for mCRC if progression is observed more than 6 months after its completion. |
|  | Reintroduction of the same treatment regimen is still considered first line if progression is observed between 3 and 6 months after its completion. |
|  | It is considered first line to reintroduce an EGFR inhibitor (anti-EGFR) with another chemotherapy regimen, in case of contraindication to the previous regimen, if progression is observed more than 6 months after the end of the previous treatment. |
| Aspects related to the use of doublets / triplets | If a patient receives treatment with a triplet chemotherapy together with a biologic agent in the first line and progresses, the next line will be considered a third line. |
|  | If a patient treated with a triplet of chemotherapy associated with a biologic agent, goes on to maintenance with 5-FU/LV + biologic agent and progresses more than 6 months after starting maintenance, reintroduction of oxaliplatin or irinotecan will be considered a second line. |
| Aspects related to maintenance | Maintenance treatment is considered as part of the first-line strategy. |
|  | If during maintenance the patient progresses and the same initial treatment regimen is reintroduced, it is still considered first line. |
|  | If during maintenance the initial therapeutic scheme is changed (e.g. change from capecitabine to 5-FU/LV or vice versa) and the patient progresses, it will be considered that two lines of treatment have been used. |
|  | During maintenance with fluoropyrimidines and anti-EGFR, anti-EGFR should be continued until disease progression, despite possible withdrawal of fluoropyrimidine. |
|  | In the absence of contraindicating toxicity, reintroduction of the same initial treatment regimen is considered indicated if disease progression occurs > 3 months after initiation of maintenance therapy. |
| Aspects related to the 2nd line approach | It is necessary to know the status of the biomarker or molecular profile in the second line to decide the therapeutic approach |
| Aspects related to the 3rd and subsequent line approach | The safety profile of the therapies to be used in the third line is crucial for the choice of treatment |
|  | The molecular profile of the tumor should be considered for the third-line approach. |
|  | In case of progression in lines prior to anti-EGFR treatment, reintroduction (rechallenge) is considered the preferred option for the third line, as long as RAS/BRAF wild-type status is confirmed. |
|  | The combination of TAS 102 and Bevacizumab is considered the preferred third-line option. |
|  | It is considered third line even if any of the previous lines was suspended due to toxicity without evidence of progression. |
|  | It is considered third line if it has not been possible to administer any of the first or second line drugs due to contraindications. |
|  | A patient who is frail or in poor general condition may receive treatments positioned in third line without having received all the previous schemes indicated in the clinical guidelines. |
